# Management of an Unsuccessful Regenerative Endodontic Procedure after Tooth Fracture: A Case Report

**DOI:** 10.3390/dj8030094

**Published:** 2020-09-01

**Authors:** Luísa Bandeira Lopes, Francisco Paredes, Andreia Pimenta, Inês Carpinteiro

**Affiliations:** 1 Pediatric Department, Centro de Investigação Interdisciplinar Egas Moniz, Egas Moniz Cooperativa de Ensino Superior, 2521-511 Caparica, Almada, Portugal; franciscomparedes@gmail.com; 2 Clinical Research Unit (CRU), Centro de Investigação Interdisciplinar Egas Moniz (CiiEM), Instituto Universitário Egas Moniz (IUEM), 2521-511 Caparica, Almada, Portugal; andreiappp491@gmail.com; 3 Dentistry Department, Centro de Investigação Interdisciplinar Egas Moniz, Egas Moniz Cooperativa de Ensino Superior, 2521-511 Caparica, Almada, Portugal; ines.m.c.carpinteiro@gmail.com

**Keywords:** regenerative endodontic procedure, tooth fracture, mineral trioxide aggregate, dental trauma

## Abstract

Dental trauma is a very frequent occurrence in children and adolescents, which creates a great impact on the esthetics, functions, and phonetics. Traumatic dental injuries can range from simple enamel fractures to permanent tooth loss. This case report presents an eight-year-old patient with an uncomplicated crown fracture of tooth 21, and 30 days after trauma, it was diagnosed as necrotic pulp. The first treatment choice was a regenerative endodontic procedure (REP), however, the failure led to apexification with Mineral Trioxide Aggregate (MTA). The chosen rehabilitation was a composite veneer. Concerning the available literature and fracture enamel dentin, the treatment approach proposed for the case provided good functional and esthetic outcomes.

## 1. Introduction

Dental trauma is one of the most common facial traumatic injuries, particularly coronary fractures and tooth luxation of permanent teeth [1].

If a non-complicated fracture occurs (without pulp exposure), tooth restoration is recommended after confirming its viability through vitality tests and radiographic exam [2]. Among the available procedures, tooth fragment reattachment is undoubtedly the best solution [2]. Nevertheless, dentin sealing with a glass ionomer or restoration is imperative to protect and preserve the pulp from external harms [2].

Overall, the risk of pulp necrosis after enamel-dentin fracture is very low [2]. Nevertheless, when exposed dentin is not detected and properly protected, it leads to a spread of oral bacteria in dentinal tubules and, therefore, triggers an inflammation chain in the underlying pulp. Other elements like the time elapsed since the injury and the distance between the surface of the fracture and the pulp, can lead to reparative or degenerative changes in the pulp, with the aggravating factor that young permanent teeth present wide dental tubules [2].

Therefore, if the irritation is not suppressed, pulp may suffer infection-related necrosis [2]. Thus, in cases associated with wide open apices, a regenerative endodontic procedure (REP) can be a treatment option [2]. The term revascularization was first introduced by por Nygaard-Ostby in 1961 [2,3]. A REP is defined as a “biologically based procedure designed to replace damaged structures including dentin and root structures as well as cells of the dentin pulp complex” [2]. Thereby, REP is a method to induce a calcified barrier in a root with an open apex or the continued apical development of an incompletely formed root in teeth with necrotic pulp [2]. REP is usually performed in two consults, where in the first appointment, aside from tooth access preparation, the root canal is disinfected and intracanal medication applied. At the second visit, intracanal medication is removed, apical bleeding is induced, Mineral Trioxide Aggregate (MTA) placed over the blood clot, and a coronal seal performed [2]. Nevertheless, clinical management of REPs can lead to unsatisfactory results, which are no apical closure and no axial or transverse growth [2]. Therefore, knowing how to overcome an unsuccessful REP is of clinical interest.

This clinical report describes an unusual case of a tooth crown fracture involving an upper central incisor after trauma associated with pulp necrosis. A REP was attempted, but without success, which was resolved later via apexification with MTA and rehabilitated with an indirect composite veneer. Finally, a four-year successful follow-up is presented. Thus, the objective is to show how important the diagnosis and the treatment plan are, and how to manage unsuccessful cases of REP.

## 2. Case Presentation

**Figure 1:**
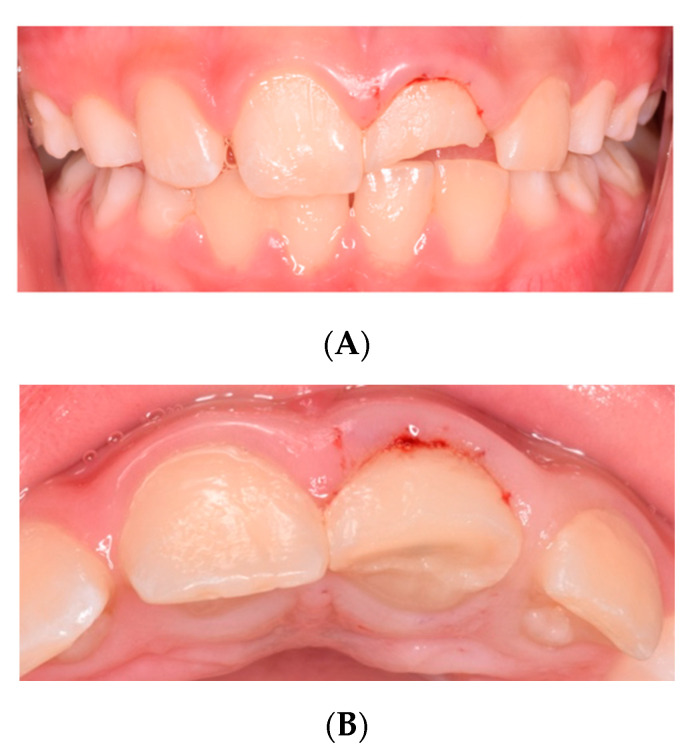
Intra-oral frontal photography. (**A**) Preoperative buccal clinical view of the anterior teeth. (**B**) Preoperative incisal clinical view of the anterior teeth.

**Figure 2:**
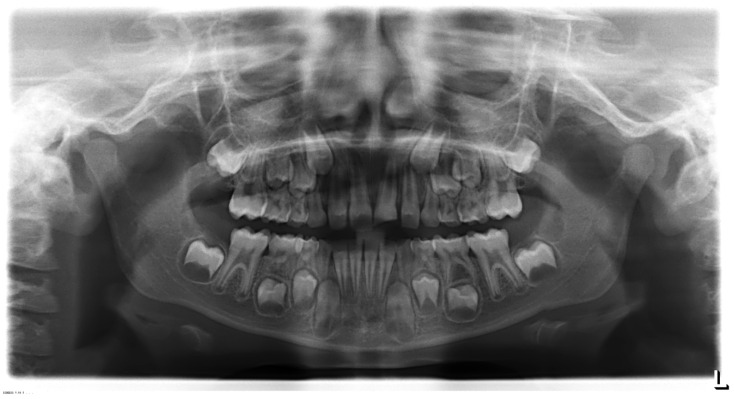
Preoperative orthopantomography.

An eight-year-old male patient presented to the pediatric department at Egas Moniz Dental Clinic, Almada Portugal for an emergency appointment 30 days after orofacial trauma. The patient had no significant medical history to report. In clinical evaluation, extraoral examination showed no facial asymmetry or swelling. Intraoral examination showed an uncomplicated crown fracture (enamel and dentin) of the upper left central incisor (21) without fistula, and soft tissue laceration or alveolar bone fracture (Figure 1). In the diagnostic test, the tooth did not show any response to the different pulp test. The radiographic examination revealed incomplete root development and absence of periapical lesion (Figure 2). Taking clinical and radiographic findings into account, the concluded diagnosis was pulp necrosis.

### 2.1. Treatment Alternatives

Three treatment options were presented to the patient’s parents. The first was apexification with MTA. However, this was rejected because it would not allow the root development. The second treatment option was an apexification procedure with calcium hydroxide (Ca(OH)_2_), where the final outcome was the same as the last, although it takes more treatment time and obliges child compliance. The third was REP. This last option was adopted since it allows the root development with apical closure and thickening of radicular dentin. Regarding the tooth rehabilitation, several options were proposed such as indirect composite veneer, direct composite veneer as well as internal bleaching due to the patient’s age. After discussion, an indirect composite veneer was planned, since it would allow a better surface texture, anatomic, occlusal stability, tooth integrity, and marginal discoloration. The treatment procedure, risks, and benefits were completely explained to the parents and then written informed consent was obtained.

### 2.2. Treatment Progress

**Figure 3:**
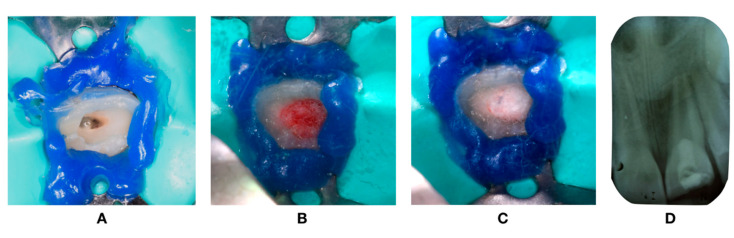
Intra-oral photography. (**A**) Coronary opening. (**B**) Blood clot. (**C**) MTA placed apical to cementoenamel junction. (**D**) Periapical radiograph after REP procedure.

At the first visit, the canal preparation was performed after local anesthesia without vasoconstrictor (3% Mepivacaine) and rubber dam isolation. Local anesthesia was delivered because of the recent history of trauma to the soft tissues. The protocol of REP with a coronary opening with a diamond bur (Figure 3A) was initiated. The root canal system was slowly irrigated with 5.25% sodium hypochlorite (NaOCl) and then 17% liquid Ethylenediaminetetraacetic acid (EDTA) in order to remove the smear layer, being both activated with dynamic manual irrigation. The EDTA was used because of the increase in stem cells of the apical papilla survival [2]. The canal was then dried, Ca(OH)_2_ (Ultracal, Ultradent, South Jordan, UT, USA) applied as an internal medication, and then sealed with temporary restoration, being Cavit G (3M ESPE, Maplewood, MN, USA).

Three weeks later, on the second visit, after clinical examination, local anesthesia without a vasoconstrictor (3% Mepivacaine) was performed and the tooth was isolated with a rubber dam. Then, the pulp chamber was accessed and the removal of intracanal Ca(OH)_2_ (Ultracal, Ultradent, South Jordan, UT, USA) was performed by saline irrigation. Followed by drying with paper points, the revascularization procedure was performed by trespassing 2 mm beyond the apical foramen with a #25K-file (Dentsply, Maillefer, Ballaigues, Switzerland) to induce bleeding. After blood clot formation (Figure 3B), MTA (ProRoot, Dentsply, Ballaigues, Switzerland) was placed apical to the cementoenamel junction (Figure 3C) and covered with resin modified glass ionomer cement (GC International AG, Luzern, Switzerland) with the objective to seal the access (Figure 3D).

**Figure 4:**
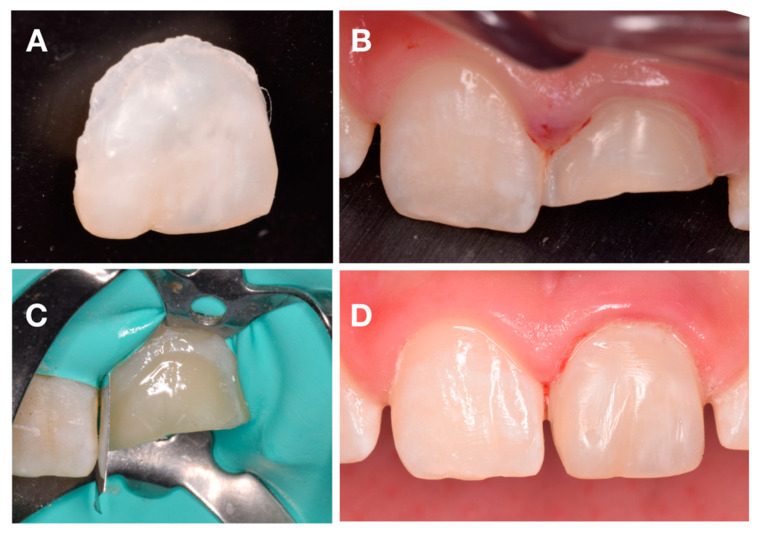
Intra-oral photography. (**A**) Composite veneer. (**B**) Veneer prep. (**C**) Rubber dam isolation. (**D**) Composite veneer immediately after cementation.

At the one-month follow-up appointment, positive sensitivity tests and negative response to percussion test were recorded. At this stage, the temporary restoration was removed, and the tooth surface was prepared for the indirect veneer with immediate dentin sealing (IDS) protocol using Optibon FL (Kerrdental, OR, USA) and Filtek Z100 (3M ESPE, Maplewood, MN, USA) on retentive areas (Figure 4A,B). Then, impressions were made using a one-step addition silicon technique Express XT and Light Body Paste (3M ESPE, Maplewood, MN, USA). on the upper arch, and in the lower arch, the impression was taken using alginate (3M ESPE, Maplewood, MN, USA).

Die stone casts were made to allow for the fabrication of the indirect composite veneer with ENA HRI UD4 (Micerium, Avegno, Italy) A4 VITA-Scale (Figure 4C).

At the following appointment, the veneer adaptation was evaluated to assess marginal fit and esthetics before the adhesive procedure. Then, after rubber dam placement, the adhesion procedure was performed according to the protocol described in Table 1. After removing the glycerin gel, the marginal area was finished and polished (Figure 4D). Restoration were checked to avoid any occlusal interference.

The patient was recalled one-month later for a follow-up visit. At this visit, the patient was asymptomatic, and the tooth showed normal response to the vitality tests and the negative response to the percussion test.

**Figure 5:**
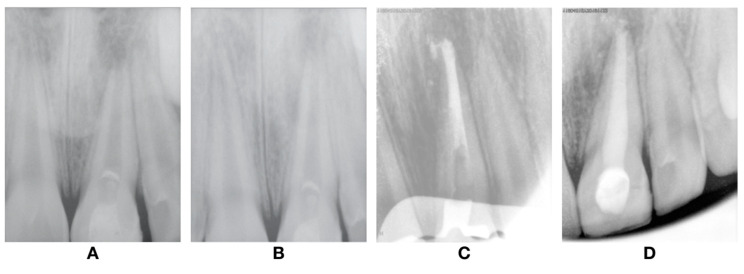
Follow-up radiographs after revascularization. (**A**) 1-year follow-up; (**B**) 4-year follow-up; and after (**C**) plug MTA; and (**D**) root canal obturation.

At one-year follow-up, clinical examination revealed no signs or symptoms of pulp injury and the soft tissues were healthy. Radiographic examination revealed no signs of dentine wall thickening or continuous root development (Figure 5A). In the two-year follow-up, the radiograph revealed evidence that there was no sign of root development and apical closure as well as dentine wall thickening (Figure 5B). Thereby, and taking the radiographic images in the last two-year findings into account, we concluded that the revascularization failed, and an alternative endodontic treatment had to be accomplished. At this stage, the treatment options presented were: (1) new attempt of the REP; (2) apexification with MTA; or (3) apexification with the Ca(OH)_2_ procedure. The apexification with MTA was the elected treatment and was performed over two visits.

**Figure 6:**
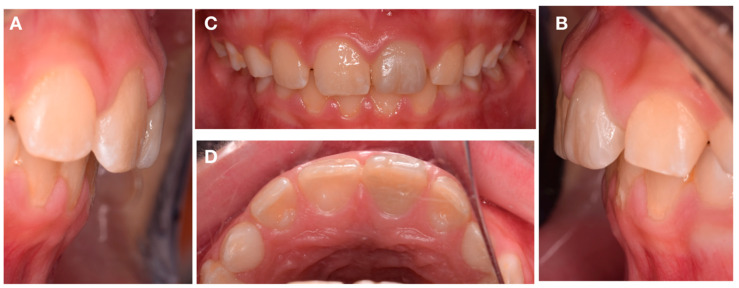
Intra-oral photography. Clinical aspect of the lateral (**A**,**B**), buccal (**C**), and palatal (**D**) view after the 4-year follow-up.

At the first visit, after the patient was anesthetized locally (2% lidocaine with 1:80,000 adrenaline) and the rubber dam placed, the restoration and the MTA were removed. Then, the root canal system was irrigated with 5.25% NaOCl and 17% liquid EDTA. The working length (18 mm) was electronically measured with a Root ZX (J. Morita) apex locator and the radiography confirmed. Later, the canals were dried using paper points and Ca(OH)_2_ (Ultracal, Ultradent, South Jordan, UT, USA) was placed into the canal. The access cavity was sealed with temporary restoration. At the second visit, three weeks later, the Ca(OH)_2_ was washed out by saline irrigation, and approximately 3 mm of MTA (ProRoot, Dentsply, Ballaigues, Switzerland) was placed from the root end (Figure 5C), and over the canal, an pulp chamber were obturated with gutta percha, system B&L (Figure 5D). Then, the access cavity was sealed and covered with a modified glass ionomer cement (GC Fugi II; GC Corporation, Japan). Finally, the endodontic access was restored with HRi/UD1 Enamel Plus (Micerium, Avegno, Italy), a high value color due to the typical MTA discoloration [4,5]. Furthermore, the veneer was polished to improve the esthetic appearance (Figure 6).

## 3. Discussion

This article reports an attempt to resolve an unsuccessful REP by the apexification with MTA. Next, we decided to apply an indirect composite veneer due to color stability [6]. This treatment option was effective until a 4-year follow-up, which allows a number of restorative management options to be considered in the future, if the esthetics is suddenly compromised.

Treating immature permanent teeth is challenging because a premature or an incorrect decision can lead to the loss of the permanent tooth. The consequences are numerous such as impairment of growth, speech, masticatory function, and esthetics [7,8,9,10].

According to the European Society of Endodontology (ESE), “pulp revitalization” is an alternative option to apexification in certain cases, since there is an increase in the scientific evidence of this approach [11]. Following this rationale, the American Association of Endodontics (AAE) states that “pulp revascularization” is the elective treatment for immature necrotic permanent teeth that have incomplete root development and open apex [12].

Although several features still remain unrecognized like the type of tissue formed as well as no protocol defined, REPs promote root development with apical closure and thickening of root dentin [12,13,14]. The first treatment choice was REP because of the possibility to foment root development with a close apex and coarsen root dentin. Despite not knowing the real reason for the failure, there are some hypotheses like the presence of bacteria in the apical part of the root. Another possibility is the disruption of the blood clot, leaving an empty space inside the canal and no proper coronal seal [2,10,12,14,15,16,17]. When a REP is confirmed as unsuccessful, alternative treatments must be considered such as apexification with MTA. Apexification is a nonsurgical procedure that promotes a calcic barrier at the open apex of nonvital teeth. This calcic barrier avoids toxins and bacteria from stepping into the peri radicular tissue and contribute to packing filling materials [9,18].

MTA presents a wide range of applications such as being an apical barrier for teeth with immature apices, repair of root perforations, root-end filling, pulp capping, and pulpotomy techniques [9]. Beyond its biocompatibility, anti-microbial activity, and marginal infiltration prevention, MTA decreases the risk of tooth fracture and coronal microleakage when compared with Ca(OH)_2_ [9,12,16].

After REP or MTA apexification, discoloration of the tooth is a common side effect and the pediatric dentist must be prepared for its management [19]. The reason for the discoloration is the presence of bismuth oxide, one of the components of MTA, which induces tooth discoloration [5]. Moreover, it is imperative to take the age of the patient into consideration, mainly because up until the age of 20, the options are limited [19,20]. Ceramic veneers are contraindicated in children and adolescents because of oral and facial development, complexity, longevity, and cost of treatment [21]. Consequently, composite veneers are a viable alternative. The main reasons are low cost, extraoral polymerization, possibility of extraoral esthetic refinement, and facilitated repair or adjustments [22]. Composite veneers have similar performance as ceramic veneers [23], though its clinical performance is difficult to compare. Notwithstanding, Meijering et al. reported no differences in the survival rate of indirect composites compared to ceramic restorations [21].

In the case that the tooth fragment is available, reattaching the original tooth fragment to restore a fractured tooth would be a very reliable option [24], since it would be more prone to better esthetics with the original tooth contours, texture and radiolucency, and function [25]. One other alternative is internal and/or external bleaching, although in some cases, the degree of shade is limited, might result in cervical resorption, and child compliance is required [19]. Despite all these limitations, bleaching and/or direct composite veneers are the most frequent treatment option in growing children [19].

On the other hand, the indirect composite technique also allows a better surface texture, anatomic, occlusal stability, tooth integrity and marginal discoloration to be achieved when compared with a direct restoration, however, studies have shown that there were no statistic differences in the performance of both techniques [26,27]. Thus, in cases of severe enamel discoloration, invasive approaches must sometimes be considered like resin restorations, ceramic veneers, and crowns later on [28].

## 4. Conclusions

REP continues to be the treatment of choice in cases of necrotic premature teeth, since it allows for root development with apical closure and thickening of root dentin, and proper crown/root proportion, though with some unsuccessful rates. Furthermore, when REP fails, there is still the possibility of apexification with MTA.

## Figures and Tables

**Table 1:** Conditioning protocol of the tooth and indirect composite veneer.

Veneer Conditioning Sequence	Tooth Conditioning Sequence
1. Sandblasting with Cojet (5 s)	1. Acid enamel and dentin etching (15 s) (37% Phosphoric Acid)
2. Rinsing and dying (30 s)	2. Rinsing and dying (30 s)
3. Acid enamel and dentin etching (15 s) (37% Phosphoric Acid)	3. Dental adhesive application (Optibond FL, Kerr, USA) without photopolymerization
4. Rinsing and drying (30 s)	4. Heated Z100 at 54 °C (3M ESPE, USA)
5. Ultrasonic vibration in distilled water (4 min)	5. Excess removal of Z100
6. Silane application in palatal indirect composite veneer	6. Photopolymerization at buccal, oral, and proximal sides (40 s each)
7. Dental adhesive application (Optibond FL, Kerr, USA) without photopolimerization	7. Glycerin gel application and photopolymerization at buccal, oral, and proximal sides (40 s each)
